# Single-stage combined cardiac and abdominal wall reconstruction in Pentalogy of Cantrell using a cemented sandwich mesh technique: a case report

**DOI:** 10.3389/fped.2026.1898905

**Published:** 2026-10-28

**Authors:** Abdul Rehman Zia Zaidi, Sara M. Samhan, Lara M. Samhan, Baraa Alghalyini, Fahad Suhayman R. Alanazi, Mohamad H. ElMahmoud

**Affiliations:** 1 Department of Family and Community Medicine, College of Medicine, Alfaisal University, Riyadh, Saudi Arabia; 2 School of Kinesiology and Health Science, York University, Toronto, ON, Canada; 3 College of Medicine, Alfaisal University, Riyadh, Saudi Arabia; 4 College of Medicine, Al-Jouf University, Sakaka, Saudi Arabia; 5 Department of Pediatric Surgery, Prince Sultan Military Medical City, Riyadh, Saudi Arabia

**Keywords:** case report, congenital heart disease, ectopia cordis, omphalocele, Pentalogy of Cantrell, single-stage repair, sternal reconstruction

## Abstract

**Background:**

Pentalogy of Cantrell (POC) is a rare congenital malformation defined by five midline defects involving the sternum, anterior diaphragm, diaphragmatic pericardium, abdominal wall, and heart. Surgical strategies vary between staged and single-stage approaches, and optimal timing remains debated. We report a case of complete POC managed with a single-stage combined cardiac and abdominal wall reconstruction at six months of age, using a cemented sandwich mesh, previously used by our team after sternal tumor resection, for the lower sternal defect.

**Case presentation:**

A six-month-old Saudi male infant, born at 38 weeks by emergency cesarean section (birth weight 1.99 kg, Apgar 8/9), was diagnosed antenatally with ectopia cordis, ventricular septal defect (VSD), and epigastric omphalocele. Amniocentesis had excluded trisomy. Postnatal echocardiography confirmed a large perimembranous VSD with inlet extension, a mid-muscular VSD, a small atrial septal defect (ASD), and an ejection fraction of 57%. A multidisciplinary team elected to proceed with a single-stage combined operation once his weight exceeded 5 kg (6.2 kg at surgery). Cardiac surgery was performed first through sternotomy (bypass time 150 min; cross-clamp time 124 min), with patch repair of the VSD and ASD and excision of a left ventricular diverticulum. The pediatric surgery team then addressed the omphalocele via midline laparotomy extension, closed the anterior diaphragmatic defect primarily, and reconstructed the lower sternal defect using gentamicin-impregnated bone cement within doubled Gore-Tex mesh (a sandwich mesh configuration). Fascial closure reached the umbilicus without clinical signs of raised intra-abdominal pressure. The patient was extubated on postoperative day three and achieved full oral feeds by day five. Echocardiography before discharge showed no residual shunt and an ejection fraction of 54%. A small incisional hernia was repaired electively about one year later, and the child remained asymptomatic at two years.

**Conclusions:**

This case demonstrates that single-stage combined cardiac and abdominal wall repair in complete POC is feasible and can reduce the burden of repeated surgical interventions. The cemented sandwich mesh achieved stable reconstruction of the lower sternal defect in this patient, offering a potential alternative for centers managing similar complex congenital anomalies.

## 1 Introduction

Pentalogy of Cantrell (POC) is a rare congenital condition first described by Cantrell, Haller, and Ravitch in 1958 as a syndrome of five midline developmental defects: a lower sternal cleft, an anterior diaphragmatic defect, a deficiency of the diaphragmatic pericardium, an epigastric omphalocele, and one or more intracardiac anomalies (1). The condition arises from defective development of the septum transversum and the adjacent lateral mesodermal folds during the third to fourth week of embryogenesis (2). Published incidence estimates vary between 1 in 65,000 and 1 in 200,000 live births, with a slight male predominance reported in some series (3, 4).

Toyama classified POC into three categories based on expression: complete (all five defects present), probable (four defects including an abdominal wall and cardiac defect), and incomplete (various combinations with fewer than four defects) (5). Ventricular septal defect is the most frequently associated cardiac anomaly; atrial septal defect, tetralogy of Fallot, and left ventricular diverticulum are also frequent (3, 6). Ectopia cordis, in which the heart protrudes partially or completely through the thoracoabdominal wall, represents the most clinically severe presentation and carries a guarded prognosis (7).

Surgical management of POC demands a coordinated multidisciplinary approach involving pediatric surgeons, cardiac surgeons, and anesthesiologists (8). Many reports describe staged repair, often addressing the midline defects first and deferring intracardiac repair (4, 9), although the sequence varies with the cardiac anatomy (10). Staging is intended to reduce hemodynamic compromise and to allow growth before cardiac intervention. Single-stage combined repair, by contrast, has been reported in selected series with favorable outcomes, particularly in patients with less complex cardiac anomalies, and may reduce cumulative surgical morbidity (11–13).

Reconstruction of the lower sternal defect in POC presents a distinct technical challenge. Several methods have been described, including primary approximation (feasible mainly in neonates), autologous rib grafts, prosthetic mesh, and acellular dermal matrix (14, 15). No single approach has emerged as a standard, and the choice often depends on the patient's age, the size of the defect, and available resources. We report a case of complete POC in a six-month-old infant managed with a single-stage combined cardiac and pediatric surgical operation. For the lower sternal defect, we used a cemented sandwich mesh (gentamicin-loaded bone cement enclosed within folded Gore-Tex mesh), a technique our team first described for chest wall reconstruction after sternal tumor resection in a young child (16). This report follows the CARE guidelines, and the completed CARE checklist is provided as Supplementary Data Sheet 1.

## 2 Case presentation

A male infant from Saudi Arabia was referred to our cardiac center at Prince Sultan Military Medical City, Riyadh, at two months of age for definitive management of antenatally diagnosed congenital anomalies. Prenatal ultrasonography at an outside facility had identified ectopia cordis, a subaortic ventricular septal defect, and an omphalocele. The mother was booked with antenatal findings of oligohydramnios and a scan suggestive of an ectopic heart; amniocentesis ruled out trisomy. The infant was delivered at 38 weeks of gestation by emergency cesarean section due to fetal distress and oligohydramnios, with a birth weight of 1.99 kg and Apgar scores of 8 and 9 at one and five minutes, respectively. At birth, an anterior abdominal wall defect with an omphalocele was noted, the lower part of the sternum was absent, and the apical cardiac pulsation was readily visible, raising the suspicion of Pentalogy of Cantrell. The infant was admitted to the neonatal intensive care unit for initial stabilization before transfer to our institution. On arrival, the omphalocele sac was intact and already covered by epithelialized skin; it was managed conservatively without dressings until the operation, with no complications.

On examination at our center, the infant weighed 2.8 kg and had no gross dysmorphic features apart from the thoracoabdominal defects. He was hemodynamically stable on Captopril and Furosemide, which had been initiated by the referring cardiologist. The anterior chest wall showed visible cardiac pulsation through a lower sternal defect, and an epigastric omphalocele was evident (Figure 1). Transthoracic echocardiography at our center confirmed a small atrial septal defect with left-to-right shunt, a large perimembranous ventricular septal defect with inlet extension, a mid-muscular ventricular septal defect, and protrusion of the left ventricle beyond the apex of the right ventricle consistent with a left ventricular diverticulum. The ejection fraction was 57%. Chest radiography and computed tomography demonstrated the sternal defect, the extrathoracic position of cardiac structures, and the omphalocele contents (Figure 1).

**Figure 1:**
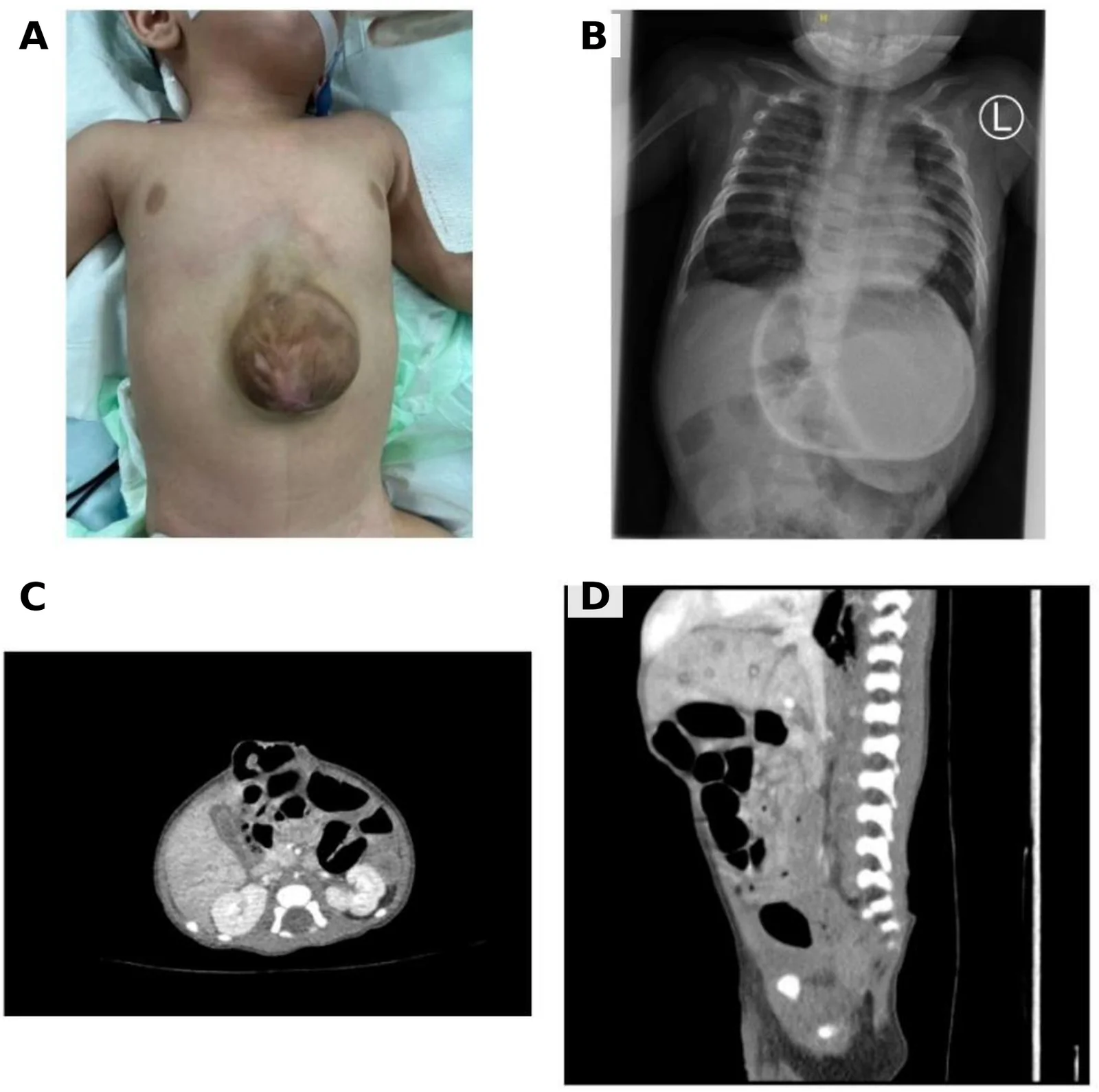
Pre-operative imaging of the patient at six months of age. **(A)** Clinical photograph showing ectopia cordis with visible cardiac pulsation through the lower sternal defect and an epigastric omphalocele. **(B)** Anteroposterior chest radiograph demonstrating the sternal defect with extrathoracic displacement of cardiac structures. **(C)** Computed tomography, axial view, showing herniation of abdominal contents through the anterior wall defect with the heart positioned outside the thoracic cavity. **(D)** Computed tomography, sagittal view, confirming the thoracoabdominal communication and the relationship of the omphalocele to the sternal defect.

The case was discussed in a multidisciplinary team meeting involving the pediatric cardiologist, cardiac surgeon, and pediatric surgeon. Given the infant's age, weight, hemodynamic stability, and the need for both cardiac and abdominal wall repair, the team elected to proceed with a single-stage combined operation rather than a staged approach. The reasoning was to avoid the cumulative risk and recovery burden of multiple general anesthetics and surgeries in a young infant with complex anatomy. Surgery was timed to coincide with the appropriate age for cardiac repair and to allow weight gain to at least 5 kg. Nutrition was optimized with oral formula feeds of 100 mL every two hours, and his weight increased from 2.8 kg at referral to 6.2 kg (length 65 cm) at the operation, which was performed at six months of age.

### 2.1 Surgical procedure

The cardiac surgery team began the operation with a median sternotomy. Cardiopulmonary bypass was established (total bypass time 150 min; aortic cross-clamp time 124 min), and the ventricular and atrial septal defects were closed with patches. The left ventricular diverticulum was excised, and the defect was repaired with 5-0 Prolene sutures buttressed with Teflon felt. The defect in the diaphragmatic pericardium was closed by covering the lower half of the heart with a Dual-mesh patch sutured to the pericardial edges and the central tendon of the diaphragm. The intracardiac repair was completed without complication; the cardiac portion of the operation lasted approximately five hours.

After completion of the cardiac portion, the pediatric surgery team extended the sternotomy incision as a midline laparotomy to the level of the umbilicus. The ventral wall defect measured 5 × 6 cm and contained small bowel and the left lobe of the liver. The contents of the epigastric omphalocele were carefully reduced into the abdominal cavity. The anterior diaphragmatic defect, which measured 3 × 5 cm, was closed primarily. The lower sternal defect, measuring about 2 × 3 cm, was then reconstructed with a cemented sandwich mesh, using the technique our team had previously applied after sternal tumor resection (16). Bone cement was prepared in the operating room by mixing its polymer powder and liquid monomer, and gentamicin was added. Gore-Tex (polytetrafluoroethylene) mesh of the type used for congenital diaphragmatic hernia repair was measured and folded to fit the defect, filled with the cement to form a sandwich, and shaped over the defect before the cement hardened. The construct was fixed with 2-0 Prolene sutures at the 12, 2, 3, 4, and 5 o'clock positions on the left and at the 8, 9, 10, and 11 o'clock positions on the right, covering the heart and bridging the sternal gap. The pediatric surgery portion lasted approximately two hours.

The midline fascia was closed from the sternum to the umbilicus using 2-0 Vicryl sutures in a continuous fashion. Intra-abdominal pressure was assessed clinically throughout closure: hemodynamic parameters did not change after closure, and urine output was monitored, although numerical values were not recorded. No signs of abdominal compartment syndrome or respiratory compromise were detected. Apart from allowing weight gain before surgery, no specific preoperative measures were taken against abdominal compartment syndrome; during and after the operation, respiratory and hemodynamic parameters and urine output were monitored continuously, together with abdominal examination. A Jackson-Pratt drain was placed subcutaneously at the laparotomy site to reduce the risk of seroma or hematoma formation.

### 2.2 Postoperative course

The patient was transferred to the cardiac intensive care unit in stable condition. He was successfully extubated on postoperative day three and tolerated gradual advancement of oral feeds, reaching full enteral nutrition by postoperative day five. The subcutaneous drain was removed on day five once output had ceased. A pericardial drain placed during the cardiac procedure was removed according to standard protocol (Figure 2). Echocardiography before discharge showed no residual ventricular or atrial septal defect, moderate tricuspid regurgitation, an estimated right ventricular systolic pressure of 28 mmHg plus right atrial pressure, good left ventricular systolic function (ejection fraction 54%), and no pericardial effusion.

**Figure 2:**
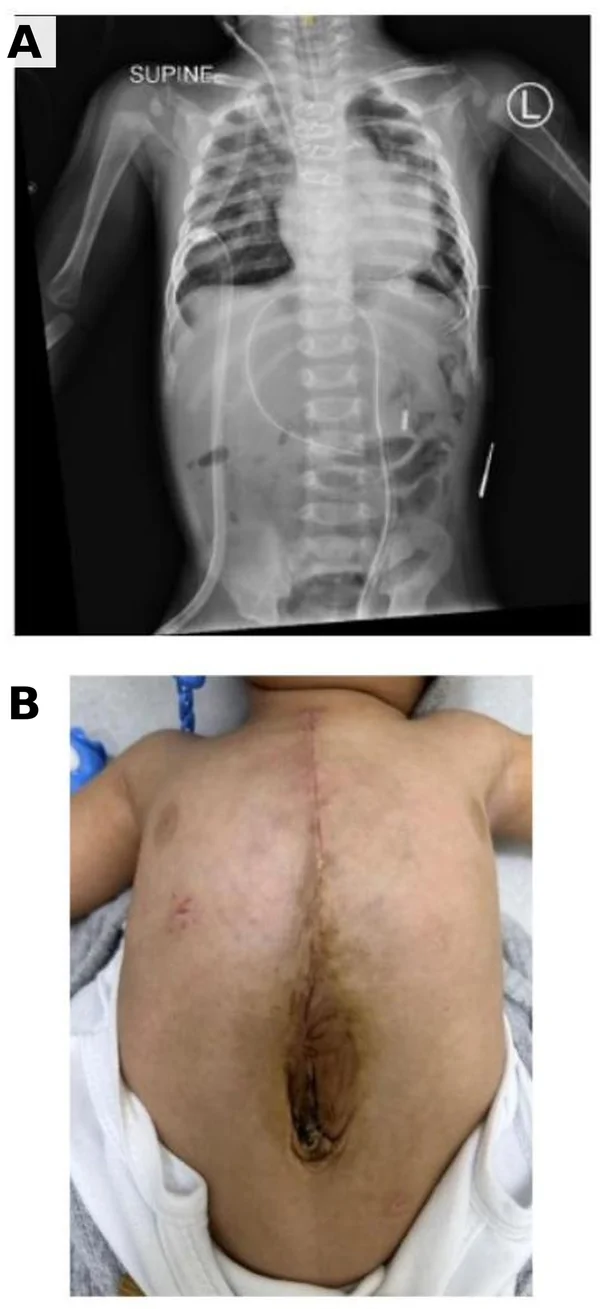
**(A)** Postoperative anteroposterior chest radiograph obtained in the immediate postoperative period, showing a subcutaneous Jackson-Pratt drain at the laparotomy site and a pericardial drain *in situ*. The cardiac silhouette demonstrates improved intrathoracic positioning following combined cardiac repair and sternal and abdominal wall reconstruction. **(B)** Clinical photograph of the abdomen obtained in the early postoperative period, showing the healing midline wound and the lower segment corresponding to the previous omphalocele repair site.

During the second postoperative week, serous discharge was noted from the lower edge of the wound near the umbilicus. Clinical examination and imaging confirmed that the fascial closure remained intact, with no evidence of dehiscence or infection. The discharge was managed conservatively with regular wound care using normal saline, and resolved prior to discharge.

At clinic follow-up, wound healing was complete. A small area of fascial defect measuring less than 5 mm was identified at the lower wound margin, at the site where serous discharge had previously occurred. This was classified as a small incisional hernia and was repaired electively about one year after the index operation, together with a circumcision. The defect then measured about 1 × 2 cm and was closed with two figure-of-eight 2-0 Vicryl sutures, and the child has remained asymptomatic since. He was last seen in clinic about 14 months after the index operation, and telephone follow-up with the family continued until just over two years after surgery without new symptoms. The follow-up clinical photograph (Figure 3) shows the healed midline wound with a satisfactory cosmetic outcome.

**Figure 3:**
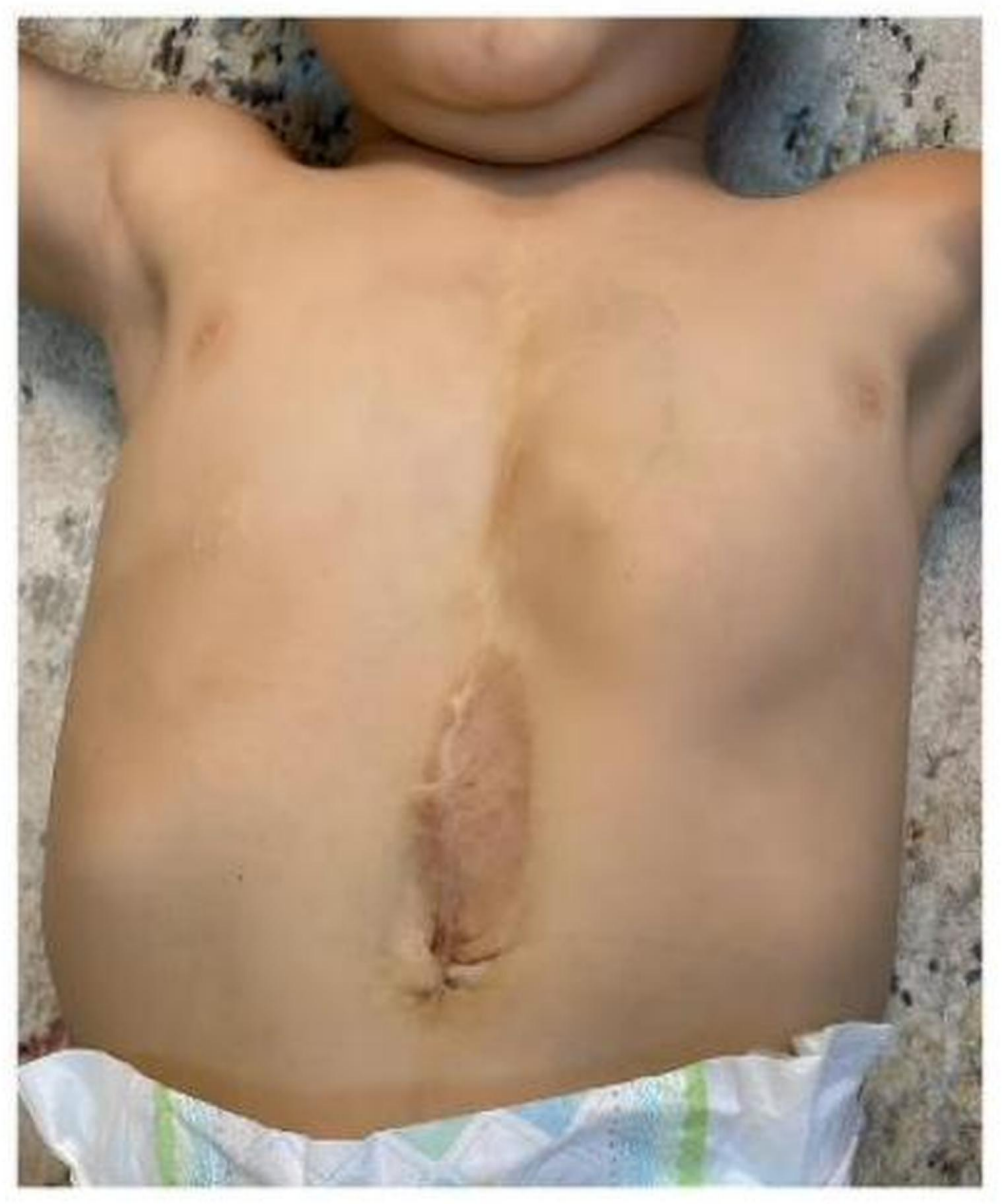
Follow-up clinical photograph obtained during outpatient review after completion of wound healing. The healed midline surgical wound extends from the sternotomy site to the umbilicus. Hypertrophic scarring is present at the lower portion, at the site of the previous omphalocele repair. The small incisional hernia at the lower wound margin (less than 5 mm) was planned for elective repair and was repaired about one year after the index operation.

## 3 Discussion

We describe the successful single-stage combined repair of all five components of Pentalogy of Cantrell in a six-month-old infant, with reconstruction of the lower sternal defect by a cemented sandwich mesh. Complete expression of POC with all five midline defects is rare; a recent unified review identified 165 well-documented cases in the global literature, with substantial heterogeneity from complete pentalogy to partial or atypical variants (17). In our patient, all five components were confirmed at operation, including the defect in the diaphragmatic pericardium, which places the case in the complete category of Toyama's classification (5). Ectopia cordis, also present in our patient, was found in 13 of 22 patients in a 42-year institutional series and was associated with higher mortality (18).

The debate between staged and single-stage surgical approaches for POC remains unresolved. A staged strategy, in which the abdominal wall defect is repaired first and intracardiac correction is deferred, has been widely described (4, 9). The theoretical advantage is that separating the procedures allows hemodynamic stabilization between operations and avoids the combined physiological stress of concurrent cardiac and abdominal surgery in a fragile infant. St. Louis reported a case of POC with hypoplastic left heart syndrome in which abdominal repair preceded cardiac palliation (9).

Single-stage repair, however, offers several practical advantages. Our decision to combine both procedures in a single session was influenced by the patient's stable hemodynamics, satisfactory ejection fraction, and the desire to limit the number of general anesthetics. Zhang and colleagues reported a series of eight patients with POC managed at a single tertiary center; seven of eight underwent one-stage complete correction with satisfactory early and midterm outcomes (12). Korver and colleagues performed early single-stage correction at 11 weeks of age, including resection of a left ventricular diverticulum (11), and Basarahalli and colleagues described a successful single-stage repair in a 17-month-old child without postoperative complications (13). Our case adds to the accumulating evidence that single-stage correction is achievable in selected patients, particularly when cardiac defects are amenable to straightforward repair and the surgical team has multidisciplinary expertise available.

An important aspect of our case is the sequence of the operation. We performed intracardiac repair first, followed by abdominal wall reconstruction. Staged reports have commonly described the reverse order, addressing the abdominal wall defect before cardiac surgery (4, 9). Our reasoning was that correcting the hemodynamically significant VSD and ASD first would provide a more stable physiological platform for the subsequent abdominal closure, which carries a risk of transiently increased intra-abdominal and intrathoracic pressures. This approach proved effective in our patient; cardiopulmonary function remained stable throughout both phases of the operation, and hemodynamic parameters did not change after abdominal closure.

Reconstruction of the lower sternal defect with a cemented sandwich mesh is a distinctive element of this report. Sternal reconstruction in infants with congenital clefts and POC has been addressed through various methods. Primary closure is preferred in neonates when chest wall compliance permits, but becomes technically difficult beyond the first few months of life as the thorax becomes more rigid (14). Autologous rib grafts, used either as single segments or split grafts, provide biological compatibility and potential for growth but add donor site morbidity (15, 19). Prosthetic mesh alone offers coverage but limited structural rigidity. In a 2021 systematic review of congenital sternal cleft reconstruction, Hinchcliff and colleagues found that 60.3% of 115 reported cases were managed with primary closure, while the remainder required interposition grafts or alloplastic materials (20). Our team first used a cement-filled Gore-Tex sandwich conduit to bridge the anterior chest wall after resection of a sternal rhabdomyosarcoma in a 2-year-old child (16), and the present case extends its use to the lower sternal defect of POC. Cementoplasty with Gore-Tex mesh on both surfaces of the cement is commonly used for pediatric chest wall reconstruction after tumor resection, and a single-side antibiotic-loaded variant in 10 children produced no reconstruction-related complications, infections, or scoliosis over a median follow-up of 5.6 years, although three children had an asymmetric chest wall appearance (21). Methyl methacrylate struts have also been used to reconstruct the anterior chest wall in ectopia cordis, with normal thoracic development at 2.5 years and follow-up extending to 10 years (22, 23). In our patient, the construct combined the rigidity of set bone cement with Gore-Tex mesh enclosing the cement on both surfaces, and it could be shaped to the defect before the cement hardened. The construct is shown schematically in Figure 4, and intraoperative images of the same configuration appear in our team's earlier report (16). Because a rigid construct may need to be revised as the chest wall grows, as our team noted after the earlier case (16), long-term follow-up of thoracic growth is needed.

**Figure 4:**
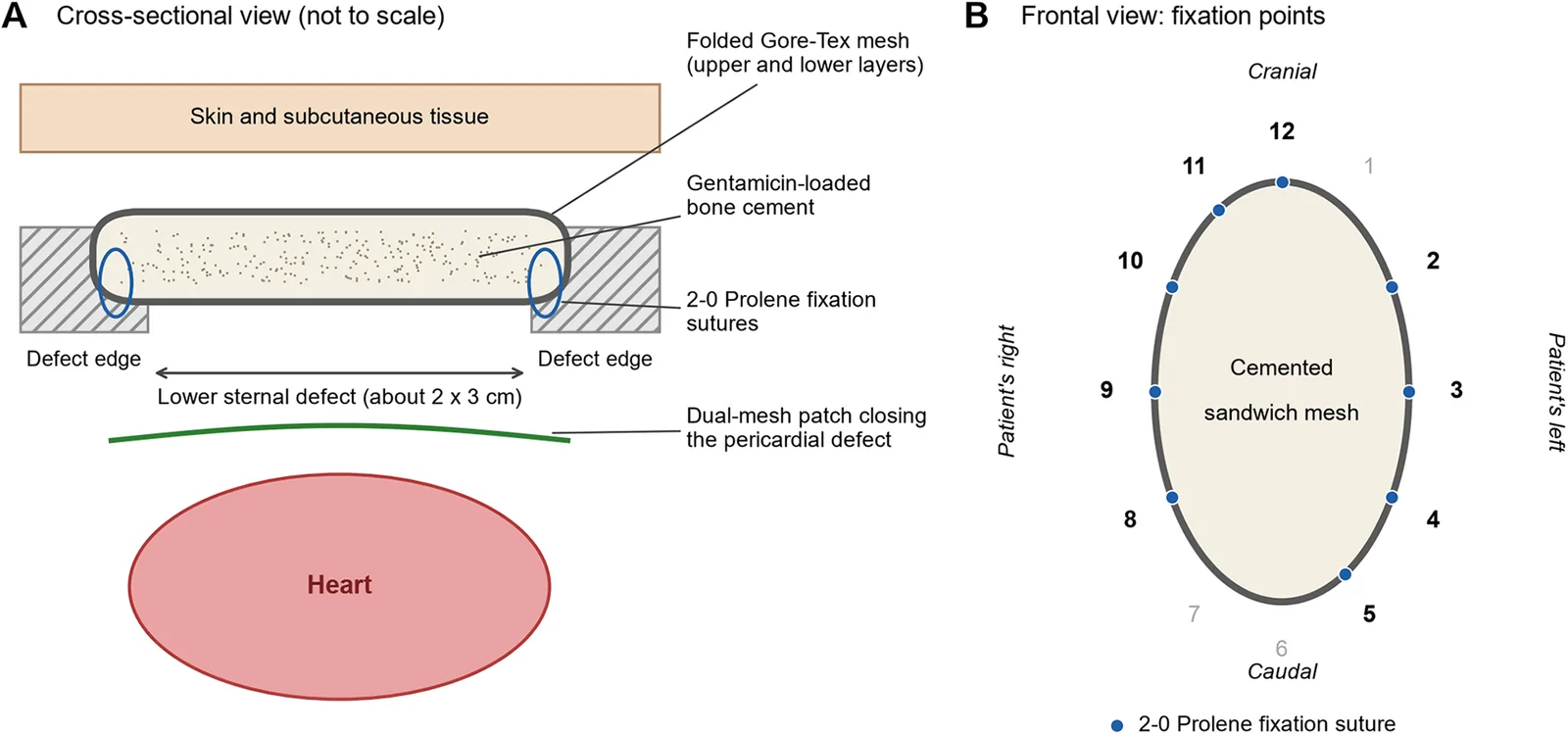
Schematic illustration of the cemented sandwich mesh (not to scale). **(A)** Cross-sectional view: gentamicin-loaded bone cement enclosed within folded Gore-Tex mesh, fixed over the heart to bridge the lower sternal defect; the defect in the diaphragmatic pericardium was closed separately with a Dual-mesh patch. **(B)** Frontal view of the 2-0 Prolene fixation points at the 12, 2, 3, 4, and 5 o'clock positions on the patient's left and at the 8, 9, 10, and 11 o'clock positions on the patient's right.

The early postoperative course in our patient was largely uneventful, in keeping with the uncomplicated courses reported after other single-stage repairs (12, 13). The serous wound discharge at the lower wound margin, at the site where a small incisional hernia later developed, likely reflects the tension at the distal end of a long midline closure in a region of inherently weakened tissue. The hernia was repaired electively with two figure-of-eight sutures about one year after the index operation, and the child has remained asymptomatic.

Prenatal diagnosis of POC, as was achieved in our patient, aids perinatal planning. Detection during the first or second trimester allows for planned delivery at a tertiary center, assembly of the surgical team, and parental counseling (24). Despite advances in prenatal imaging and surgical technique, overall mortality in complete POC remains high, particularly when severe cardiac anomalies such as single-ventricle physiology or complex outflow tract malformations are present (8, 18). Cases with less complex cardiac lesions, such as the septal defects in our patient, carry a more favorable prognosis and are generally more amenable to definitive surgical correction (12). Survival into adulthood with ectopia cordis is exceptional and depends on the severity of the intracardiac and thoracoabdominal defects (25).

Several limitations of this case merit acknowledgment. As a single case report, it cannot establish the superiority of the sandwich mesh technique or of single-stage repair over alternative approaches. Intra-abdominal pressure was assessed clinically, and numerical hemodynamic and urine output values were not available; the size of the left ventricular diverticulum was not documented; and the latest echocardiogram available to us was obtained before discharge. Clinic follow-up extended to about 14 months after surgery, with telephone follow-up thereafter, and structured patient- or parent-reported outcome measures were not collected. Long-term data on sternal growth and the durability of the construct are not yet available. Controlled comparative studies, though challenging given the rarity of POC, would be needed before drawing firm conclusions about optimal surgical strategy.

## 4 Conclusion

We present a case of complete Pentalogy of Cantrell in a six-month-old infant managed successfully with a single-stage combined cardiac and abdominal wall reconstruction. The cemented sandwich mesh provided adequate structural support for the lower sternal defect, and the child remained asymptomatic at just over two years of follow-up. This case supports the feasibility of single-stage repair in selected patients with stable hemodynamics and correctable cardiac defects, and adds the cemented sandwich mesh to the options for sternal reconstruction in congenital midline defects.

## Data Availability

The original contributions presented in the study are included in the article/supplementary material, further inquiries can be directed to the corresponding author.
