# Supplementary material for: Single-stage combined cardiac and abdominal wall reconstruction in Pentalogy of Cantrell using a cemented sandwich mesh technique: a case report
Source: Front Pediatr. 2026 Oct 28;14:1898905. doi: 10.3389/fped.2026.1898905 (PMC13752133; doi:10.3389/fped.2026.1898905)
Supplement: Supplementary file 1 [file Datasheet1.docx]

**CARE Checklist**

*CAse REport Guidelines*

**Single-Stage Combined Cardiac and Abdominal Wall Reconstruction in Pentalogy of Cantrell**

**Using a Cemented Sandwich Mesh Technique: A Case Report**

| **Topic** | **CARE Checklist Item** | **Reported on Line/Section** |
| --- | --- | --- |
| **Title** | The diagnosis or intervention of primary focus followed by the words 'case report' | Title, Page 1 |
| **Keywords** | 2 to 5 key words that identify diagnoses or interventions in this case report, including 'case report' | Page 1 (after Abstract) |
| **Abstract** | Introduction: What is unique and why is it important? | Abstract, Background |
| **Abstract** | The patient's main concerns and important clinical findings | Abstract, Case Presentation |
| **Abstract** | The main diagnoses, therapeutic interventions, and outcomes | Abstract, Case Presentation |
| **Abstract** | Conclusion: What are the main 'take-away' lessons? | Abstract, Conclusions |
| **Introduction** | Brief background summary of this case referencing the relevant medical literature | Introduction, paragraphs 1 to 4 |
| **Patient Info** | De-identified patient specific information | Case Presentation, Paragraph 1 |
| **Patient Info** | Primary concerns and symptoms of the patient | Case Presentation, Paragraph 1 |
| **Patient Info** | Medical, family, and psychosocial history including genetic information | Case Presentation, paragraph 1 (prenatal findings, oligohydramnios, amniocentesis excluding trisomy, birth at 38 weeks); further family and psychosocial history not available |
| **Patient Info** | Relevant past interventions with outcomes | Case Presentation, paragraphs 1 to 3 (NICU care, conservative management of the epithelialized omphalocele, captopril and furosemide, preoperative nutrition) |
| **Clinical Findings** | Describe the relevant physical examination (PE) findings | Case Presentation, paragraph 2 (weight 2.8 kg at referral, visible cardiac pulsation, omphalocele, no dysmorphic features); Surgical Procedure, paragraph 2 (defect dimensions) |
| **Timeline** | Historical and current information ordered chronologically | Case Presentation and Postoperative Course (antenatal diagnosis, referral at 2 months, surgery at 6 months, hernia repair about 1 year later, follow-up to just over 2 years) |
| **Diagnostic Assessment** | Diagnostic methods (PE, laboratory testing, imaging, questionnaires) | Case Presentation, Paragraph 2 (echo, CXR, CT) |
| **Diagnostic Assessment** | Diagnostic challenges | Case Presentation, paragraph 1 (antenatal diagnosis at an outside facility); Discussion, paragraph 1 (pericardial defect confirmed at operation) |
| **Diagnostic Assessment** | Diagnostic reasoning including other diagnoses considered | Discussion, paragraph 1 (all five components confirmed; complete POC in Toyama's classification); Case Presentation, paragraph 1 (amniocentesis excluding trisomy) |
| **Diagnostic Assessment** | Prognostic characteristics where applicable | Case Presentation, paragraph 3 (ejection fraction, hemodynamic stability, weight); Discussion, paragraph 7 |
| **Therapeutic Intervention** | Types of intervention (pharmacologic, surgical, preventive) | Surgical Procedure; Case Presentation, paragraph 3 (preoperative nutrition) |
| **Therapeutic Intervention** | Administration of intervention (dosage, strength, duration) | Surgical Procedure (bypass 150 min, cross-clamp 124 min, septal patches, diverticulum excision with 5-0 Prolene over Teflon felt, Dual-mesh pericardial patch, gentamicin-loaded bone cement in folded Gore-Tex mesh, 2-0 Prolene fixation, 2-0 Vicryl fascial closure); Figure 4 |
| **Therapeutic Intervention** | Changes in therapeutic interventions with rationale | Surgical Procedure; Discussion, paragraph 4 (cardiac-first sequence) |
| **Follow-up and Outcomes** | Clinician-assessed and patient-assessed outcomes | Postoperative Course, paragraphs 1 and 3 (extubation day 3, full feeds day 5, follow-up to just over 2 years); structured patient-reported outcomes not collected (Discussion, paragraph 8) |
| **Follow-up and Outcomes** | Important follow-up diagnostic and other test results | Postoperative Course, paragraph 1 (pre-discharge echocardiography: no residual shunt, moderate tricuspid regurgitation, ejection fraction 54%) |
| **Follow-up and Outcomes** | Intervention adherence and tolerability | Postoperative Course, paragraphs 2 and 3 (clinic follow-up to about 14 months, telephone follow-up thereafter) |
| **Follow-up and Outcomes** | Adverse and unanticipated events | Postoperative Course, paragraphs 2 and 3 (serous wound discharge; incisional hernia repaired about 1 year after surgery) |
| **Discussion** | Strengths and limitations of the management of this case | Discussion, paragraphs 1 to 8 (limitations in paragraph 8) |
| **Discussion** | Relevant medical literature with references | Discussion (25 references cited throughout) |
| **Discussion** | The rationale for conclusions | Discussion, final paragraphs + Conclusion |
| **Discussion** | The primary 'take-away' lessons of this case report | Conclusion |
| **Patient Perspective** | Patient perspective on treatment (when appropriate) | Not available (infant; structured parent-reported outcomes not collected; stated in Discussion, paragraph 8) |
| **Informed Consent** | Patient informed consent documented | Declarations (written informed consent for publication obtained from the parents on 15 April 2026) |

*This case report was prepared in accordance with the CARE (CAse REport) guidelines.*

*Reference: Gagnier JJ, Kienle G, Altman DG, et al. The CARE guidelines: consensus-based clinical case reporting guideline development. Headache. 2013;53(10):1541-1547.*
